# *Pleurotus pulmonarius* Strain: Arsenic(III)/Cadmium(II) Accumulation, Tolerance, and Simulation Application in Environmental Remediation

**DOI:** 10.3390/ijerph20065056

**Published:** 2023-03-13

**Authors:** Yuhui Zhang, Xiaohong Chen, Ling Xie

**Affiliations:** 1 Horticulture College, Hunan Agricultural University, Changsha 410128, China; 2 Hunan Engineering Research Center of Edible Fungi, Changsha 410128, China; 3 Key Laboratory for Vegetable Biology of Hunan Province, Changsha 410128, China; 4 Engineering Research Center for Horticultural Crop Germplasm Creation and New Variety Breeding, Ministry of Education, Changsha 410128, China

**Keywords:** *Pleurotus pulmonarius*, arsenic, cadmium, accumulation, tolerance

## Abstract

The arsenic (As, III) and cadmium (Cd, II) accumulation and tolerance traits of a new strain *Pleurotus pulmonarius* MT were evaluated, and the utilization of the strain for repairing contaminated liquid and soil was explored. The hypha cultivated in potato dextrose agar (PDA) exhibited medium or high Cd accumulation (0 to 320 mg/L), medium Cd tolerance (maximum tolerated concentration, MTC ≥ 640 mg/L), medium As accumulation (0 to 80 mg/L), and high As tolerance (MTC > 1280 mg/L). The hypha has application potential in processes related to the removal of Cd and As in aqueous pollutants at concentrations of 80 mg/L Cd and 20 mg/L As. The trends obtained for the fruiting bodies of *P. pulmonarius* MT seemed to deviate from those of the hypha of this strain. The results show that the fruiting bodies featured medium As accumulation (0 to 40 mg/kg), medium As tolerance (MTC > 160 mg/kg), medium Cd accumulation (0 to 10 mg/kg), and high Cd tolerance (MTC > 1280 mg/kg). The fruiting bodies of *P. pulmonarius* MT were utilized in processes related to the recovery of Cd and As in substrates, that is, 12% contaminated soil mixed with 50 mg/kg Cd and 200 mg/kg As; thus, the hypha and fruiting bodies of *P. pulmonarius* MT can be used for the decontamination of water and soil containing As(III) and Cd(II).

## 1. Introduction

The issue of soil and water pollution has been a persistent concern for humans, leading to adverse and long-lasting impacts. More than 20 million hectares of soil have been contaminated worldwide by heavy metals (HMs) and other metalloid pollutants [1]. Water pollution accounts for deaths of more than 14,000 people daily and 1.6 million children each year worldwide [2,3]. Rapid global industrial development, together with the long-term use of fertilizers and pesticides have led to a significantly increased risk of environmental contamination with HMs [4,5]. Some non-essential HMs such as cadmium (Cd), mercury (Hg), and silver (Ag), which are toxic and useless to plants, can adversely affect the soil quality and reduce crop production [6]. These pollutants are also major causes of life-threatening degenerative diseases affecting humans such as cancer, liver injury, and immune-related and inflammatory diseases [7,8,9,10]. Among these above, Cd is the most widespread and dangerous for living organisms; it reduces plant growth or causes plant death [11] and causes human diseases such as kidney disease and several cancers [12]. As a metalloid, arsenic (As) also poses a significant toxicity threat to plants, humans, and animals, given that it is a food chain contaminant [13]. The removal of toxic trace elements (TTEs)from the environment has become a major issue, and removal technologies have received increasing attention.

Biosorption is the ability of biological materials to remove TTEs through metabolic mediation or absorption. It has several inherent advantages, including a more complete removal, lower economic cost, higher feasibility, and higher safety, different from traditional methods, including chemical precipitation, membrane separation, and ultrafiltration [14]. Biosorption mainly consists of phytoremediation [15] and mycoremediation [16]. Compared with phytoremediation, a plant-based technology for the removal of TTEs, mycoremediation (bioremediation using fungi) is a more promising method because of its low cost, short remediation period, and high efficiency [17,18]. Mycoremediation also has limitations such as its sensitivity to high levels of humidity as well as extremely high or low temperatures.

Several studies reported the outstanding accumulation of high levels of Cd, As, and other TTEs by fungi such as *Agaricus brasiliensis* [19], *Pleurotus ostreatus* [20,21], *Lentinula edodes* [22], *Trichoderma* [23], and *Phanerochaete chrysosporium* [24]. Some reports indicated that a microorganism strain normally featured capacities of high accumulation and low tolerance, low accumulation and high tolerance, or low accumulation and low tolerance [19,25]. It is a slight possibility that these microorganisms will be applied for TTEs removal until researchers find a microorganism strain that simultaneously has high accumulation and medium tolerance capacities or medium accumulation and high tolerance capacities. In most cases, two or more kinds of TTEs always exist in environmental pollutants. It is necessary to explore some efficient techniques or fungi for removing two or more kinds of TTEs. Furthermore, most studies on the association between fungi and HMs pollution only investigated a specific stage of fungal growth (mycelia or fruiting body stages), such as *P*. *ostreatus* [21] and *L*. *edodes* [22]. Consequently, the applicability of mycelial TTEs stress patterns to the fruiting bodies of the same fungal strain remains uncertain. This creates practical limitations, particularly in simulated soil experiments, due to the sensitivity of macro fungi to high soil content. While some reports on the use of *P. leurotus* sp. such as *P*. *ostreatus* [21] and *P*. *eryngii* [26] for micro-mediation exist, the potential of *P. pulmonarius* in this domain requires further investigation. Our preliminary experiments indicated that the *P. pulmonarius* MT strain we collected in the wild could thrive in the substrate containing significant amounts of soil, increasing the likelihood of success in our simulated soil experiments. Our current study deals with evaluation of accumulation and tolerance to Cd (II) and As (III) in hypha and fruiting bodies of *P. pulmonarius* MT, and mycoremediation feasibility for removal of Cd and As by simulated soil experiments.

## 2. Materials and Methods

### 2.1. Strain, Chemicals, Medium, and Substrates

The *P. pulmonarius* MT strain (CCTCC M 2021011) obtained via tissue isolation from an abandoned cultivation base for edible mushrooms contaminated with HMs (Changsha, Hunan Province, China) was used in this study. This strain was stored in the China Center for Type Culture Collection, Wuhan University, Wuhan, China, and has been filed for Chinese patent application (application no. 202110040646.2, State Intellectual Property Office of P. R. China). The hypha was initially incubated at 25 °C for 2 weeks in potato dextrose agar (PDA) containing 12 g/L potato extract, 20 g/L glucose, and 20 g/L agar (Solarbio, China).

Furthermore, 99.4% wheat grains boiled in water for 0.5 h, 0.3% calcium carbonate (CaCO_3_), and 0.3% calcium sulfate (CaSO_4_) (*w*/*w*) were mixed as substrates for strain cultivation. Substrates were packed in glass bottles, and 6 mm hypha blocks were transplanted on the substrates. The cultivation strain was incubated at 25 °C until the grains were completely covered with hypha.

Deionized water was used during all experiments. The As and Cd concentrations were controlled in PDA, potato dextrose broth (PDB), wheat grains, cottonseeds hull, corncob, and unpolluted soil (As ≤ 1 ppm; Cd ≤ 1 ppm; dry weight).

### 2.2. Preparation of HM Salts Solutions

CdCl_2_ and NaAsO_2_ were pre-dried to achieve constant weight, and then 8.15 g and 8.67 g of CdCl_2_ and NaAsO_2_, respectively, were dissolved in 100 mL of deionized water. Then, 50 g/L of Cd (II) and As (III) solutions were prepared and stored at 4 °C.

### 2.3. Cd (II) or As (III) Stress Treatment for Hypha

The hypha of *P. pulmonarius* MT under Cd stress was monitored on a PDA medium supplemented with different Cd concentrations (Cd^2+^: 0, 80, 160, 320, 640, 1280, and 2560 mg/L). Under As stress, there were other seven groups of different As concentrations (As^3+^: 0, 80, 160, 320, 640, 1280, and 2560 mg/L^)^. Each treatment was conducted four times. The addition methods for As were the same as those for Cd. Hypha block (6 mm) was then inoculated on the PDA of every plate and incubated for 2 weeks at 25 °C. After 14 days, the colony diameter was measured, and the hypha inhibition rate (HIR) was calculated. The hypha was harvested from the PDA surface, dried at 60 °C for 24 h, and ground. Approximately 0.1 g of dry samples was collected and then digested with 7 mL HNO_3_ and 1.5 mL H_2_O_2_ via a microwave digestion system before inductively coupled plasma–mass spectrometry (ICP-MS, Agilent 7900, Waldbronn, Germany) analysis. The process for the solid medium after incubation was the same as that of the hypha above, with drying at 105 °C for 24–36 h, and 0.2 g of dry samples was collected. The As or Cd concentrations in all samples were determined via ICP-MS [27]. The calibration range was 0–100 μg/L for As and Cd.

HIR (%) = (*W_c_* − *W_a_*)/*W_c_* × 100%, where *W_c_* (g) represents the weight of the fresh hyphae in the control (CK) group; and *W_a_* (g) represents the weight of the fresh hyphae cultivated under TTEs stress.

### 2.4. Remediation Experiment of Contaminated Liquid with Cd and As

Different concentrations of an artificially contaminated liquid medium with Cd and As (0 and 0, 80 and 20, 160 and 40, 20 and 80 mg/L) were separately obtained after PDB was mixed with As and Cd solutions. Each treatment was conducted for four repetitions in 250 mL flasks with 100 mL liquid.

After four fungi blocks (6 mm) were added into the liquid, the mycelia of *P. pulmonarius* MT was monitored in PDB supplemented with different concentrations of Cd and As for 2 weeks at 25 °C and 120 r/min. After 14 days, the mycelia was filtered, collected, washed with deionized water three times, dried at 60 °C for 24 h, and weighed as biological yield. Then, 5 mL samples of the liquid medium before and after incubation were separately collected, vaporized to obtain dry samples, and then prepared as mycelia under Cd or As stress. The Cd and As amounts were analyzed via ICP-MS.

### 2.5. Cd or As Stress Treatment for Fruiting Bodies and Cultivation Management

First, 85% cotton seeds hull (dw), 12% corncob (dw), and 3% CaCO_3_ (*w*/*w*) were mixed as cultivation substrates for the fruiting bodies of *P. pulmonarius* MT. The fruiting bodies under Cd stress were monitored on the substrates above supplemented with different concentrations (Cd^2+^: 0, 5, 10, 20, 40, 80, 160, 320, 640, and 1280 mg/kg). Under As stress, there were 10 other groups of different concentrations (As^3+^: 0, 5, 10, 20, 40, 80, 160, 320, 640, and 1280 mg/kg). Each treatment was conducted for four repetitions. The detailed procedure is as follows: A total weight of 3 kg substrate ingredients from one treatment was placed in a porcelain container, mixed with about 300 mL diluents of Cd for pre-wetting until relative humidity of 55–65%. The lack of liquid for pre-wetting was replaced with deionized water. The pH values of the substrates were adjusted to 7–7.5. The addition strategies for As were the same as that for Cd.

The experimental site was the Hunan Engineering Research Center of Edible Fungi. Substrates were filled into polyethylene plastic bags (17 cm × 33 cm × 0.045 cm) and sterilized at 121 °C for 2 h. After the substrates were cooled, they were inoculated in each bag with 10 g hypha from bottles as described in the second paragraph of Section 2.1. Incubation was conducted at 25 °C and 80–85% (air humidity) for 20–30 days. At the fructification phase, cultivation was conducted at a temperature of 24–26 °C, relative air humidity of 90–95%, and CO_2_ of 2000 ppm. Harvesting was usually performed early in the morning before spore ejection after another 20–30 day cultivation. As the first batch normally accounts for about 50% of the total biological yield, the fruiting bodies of the first batch harvested were only weighed after drying at 60 °C for 24–48 h, and they were used for biological yield (g, dw) calculation and ICP-MS determination. Moreover, 0.2 g fruiting bodies, substrates before cultivation, and substrates after cultivation were separately prepared via the same approach as above before the Cd or As amount was evaluated via ICP-MS. All the samples were dry.

### 2.6. Remediation Experiment of Cd- and As-Contaminated Soil

First, 5 g/L of Cd solution was obtained after 16 mL, 50 g/L of Cd solution was mixed with 144 mL deionized water. Then, 1.25 g/L of As solution was also obtained after 4 mL, 50 g/L of As solution was mixed with 156 mL deionized water. Then, 4 kg of artificially contaminated soil (pH = 6.7) with 50 mg/kg of Cd and 200 mg/kg of As was obtained after the soil was completely mixed with two diluted solutions as described in Section 2.2. After the contaminated soil was dried at 105 °C for 48–72 h, it was collected and stored in a dry environment. Except for the CK group, four groups of substrates were mixed with different proportions of the contaminated soil described above before being filled into plastic bags. The proportions of contaminated soil were 12%, 24%, 36%, and 48%. The cultivation process was the same as that for the fruiting bodies above. In this experiment, the whole cultivation time was about 90 d. The preparation process for all samples was the same as that for the fruiting bodies above. The Cd and As amounts were analyzed via ICP-MS.

### 2.7. Statistical Analysis

Statistical analysis was performed via analysis of variance. The Statistical Package for Social Sciences 18.0 software package (USA) was used to perform statistical analysis, and differences with *p* values of ≤0.05 were considered statistically significant.

## 3. Results

### 3.1. Effect of Cd Stress on the Hypha of P. pulmonarius MT

**Figure 1:**
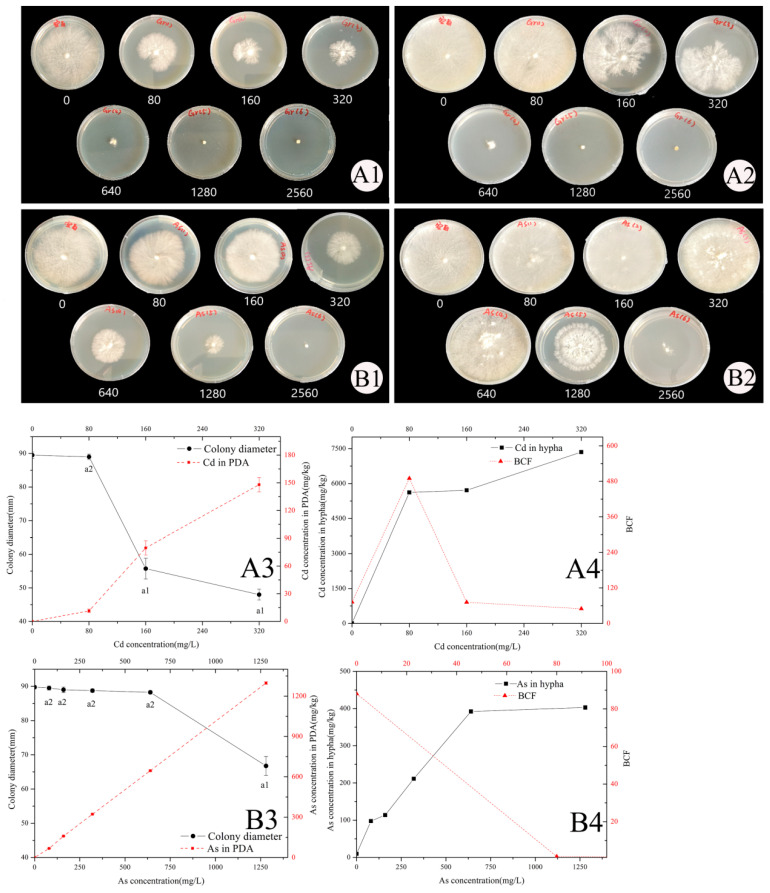
Effect of Cd and As stress on colony diameter and Cd/As content of the hypha in *P. pulmonarius* MT (X ± s, n = 4). (**A1**) hypha incubated 7 d under Cd stress; (**A2**) hypha incubated 14 d under Cd stress; (**B1**) hypha incubated 7 d under As stress; (**B2**) hypha incubated 14 d under As stress; (**A3**) colony diameter and Cd content in PDA; (**A4**) Cd content in hypha and bio-enrichment coefficients (BCF); (**B3**) colony diameter and As content in PDA; (**B4**) As content in hypha and bio-enrichment coefficients (BCF); a1: Each group was compared to CK group and two groups were significantly different (*p* < 0.05); a2: Each group was compared to CK group and two groups were not significantly different (*p* > 0.05).

The results of Figure 1(A1–A4) reveal the effect of Cd at the tested levels for the hypha of *P. pulmonarius* MT. When the concentration of Cd stress in PDA is 80 mg/L, the colony diameter of the hypha cultivated for 2 weeks is slightly lower than that for control group (CK) (*p* > 0.05). This shows that the presence of ≥160 mg/L Cd significantly inhibits the colony diameter on agar (*p* < 0.05). Under the maximum tolerated concentration (MTC), the hypha growth is completely inhibited, and the HIR is about 93% (89–6 mm/89 mm). Therefore, the MTC of Cd for *P. pulmonarius* MT in PDA is ≥640 mg/L.

Although no significant difference in hypha growth is observed between CK and 80 mg/L treatment, the Cd content in hypha rapidly rises from 5.78 to 5619.62 mg/kg. The colony diameter for 160 mg/L treatment is reduced to 55.75 mm (*p* < 0.05), while the Cd content in hypha is enhanced up to 5712.77 mg/kg. The 320 mg/L treatment gives the highest values (7354.61 mg/kg) for hypha, and the 80 mg/L treatment gives the highest bio-enrichment coefficients (BCF, 418–591).

### 3.2. Effect of As Stress on the Hypha of P. pulmonarius MT

When the As concentration ranges from 80 to 640 mg/L in PDA, the colony diameter is slightly lower than that of the CK group (*p* > 0.05) (Figure 1(B2,B3)). The results illustrate that the presence of ≥1280 mg/L As significantly inhibits the hypha growth on agar plates (*p* < 0.05). Moreover, the MTC of As for *P. pulmonarius* MT in PDA is >1280 mg/L.

The As content in the hypha increases from 9.69 to 402.87 mg/kg with increasing As concentration in the PDA (0–1280 mg/L) (Figure 1(B4)). For the same concentration of As treatment, the As content in the hypha is lower than that in the PDA after incubation, except for CK and 80 mg/L treatments. In other words, only *P. pulmonarius* MT under a low concentration of As stress (≤80 mg/L) can transfer more As ions from PDA into the hypha and shows a trait of low As accumulation (BCF, 1.4–1.5) or medium As accumulation (BCF, 66.9–125).

### 3.3. Remediation Effect of P. pulmonarius MT on Cd- and As-Contaminated Liquid

**Figure 2:**
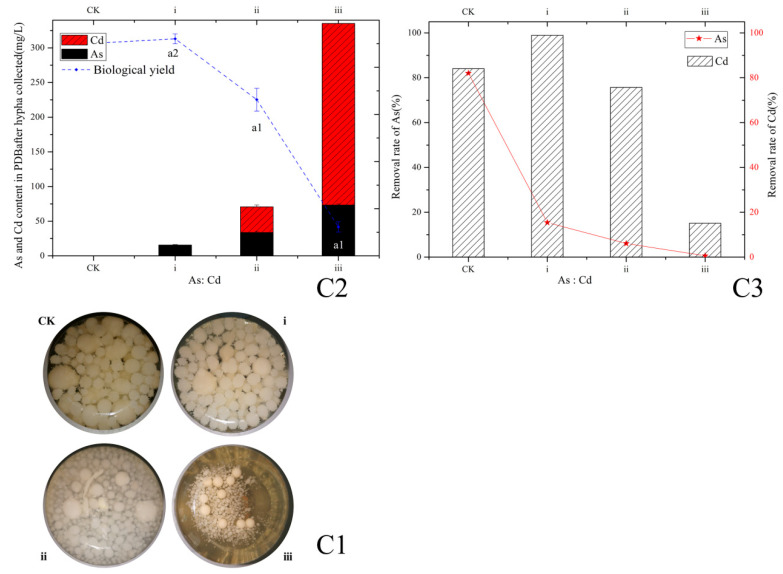
Simulation application in decontamination of liquid containing As(III) and Cd(II) by the hypha of *P. pulmonarius* MT. (**C1**) Mycelia growth under Cd and As stress; (**C2**) Cd&As content in PDB and the biological yield of mycelia under Cd &As stress; (**C3**) Removal rates of Cd and As. CK: Untreated PDB medium; (i) PDB with 20 mg/L of As and 80 mg/L of Cd; (ii) PDB with 40 mg/L of As and 160 mg/L of Cd; (iii) PDB with 80 mg/L of As and 320 mg/L of Cd; a1: *p* < 0.05; a2: *p* > 0.05.

Based on the traits of medium or high Cd accumulation (0 to 320 mg/L) and medium Cd tolerance (MTC ≥ 640 mg/L) in hypha cultivated in PDA and medium As accumulation (0 to 80 mg/L) and As high tolerance (MTC >1280 mg/L), the hypha of *P. pulmonarius* MT was used to decontaminate liquid polluted with As and Cd. With the increase in the As and Cd concentrations in PDB to 20 and 80 mg/L, the Cd removal rate is about 98.99% (Figure 2(C3)); the As is partly removed by 15.43%; and the mycelia growth is not significantly inhibited (*p* > 0.05, Figure 2(C1,C3)). Meanwhile, the Cd content detected in PDB for treatment is 0.77 mg/L after incubation (Figure 2(C2)). Therefore, the Cd concentration in contaminated liquid (1–80 mg/L Cd) can reach the effluent standard after treatment using *P. pulmonarius* MT. Moreover, the Cd removal rate for treatment (ii) is about 75.71%, and As is partly removed (Figure 2(C3)), although the mycelia growth is significantly inhibited (*p* < 0.05). Also, compared to the CK treatment, the size of hypha pellets for treatments (ii) and (iii) (Figure 2(C1)) is reduced and rough selvedge disappears, which is related to the toxic effect of As and Cd.

### 3.4. Effect of Cd Stress on Fruiting Bodies of P. pulmonarius MT

**Figure 3:**
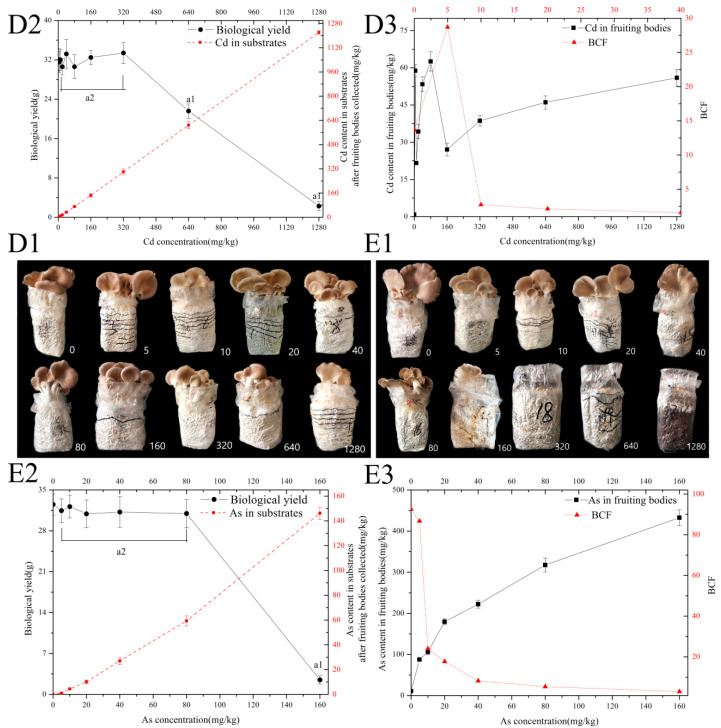
Effect of Cd and As stress on biological yield and Cd/As content of the fruiting bodies in *P. pulmonarius* MT (x ± s, n = 4). (**D1**) fruiting bodies cultivated under Cd stress; (**D2**) biological yield of fruiting bodies and Cd content in substrate; (**D3**) Cd content in fruiting bodies and bio-enrichment coefficients (BCF); (**E1**) fruiting bodies cultivated under As stress; (**E2**) biological yield of fruiting bodies and As content in substrate; (**E3**) As content in fruiting bodies and BCF; a1: *p* < 0.05; a2: *p* > 0.05.

The rules applied for the hypha of *P. pulmonarius* MT under TTEs stress were not the same as those for the fruiting bodies of this strain. It seems that the As and Cd accumulation and tolerance in fruiting bodies differ from those in hypha in our study. As shown in Figure 3(D3), with Cd doses increased from 0 to 40 mg/kg in substrates, the BCF of Cd increases to 25.5–32.4 and then reduces to 1.4–1.8. Moreover, there is no significant difference in biological yield between any ≤320 mg kg^−1^ treatment and CK (*p* > 0.05, Figure 3(D1,D2)). Regarding the Cd tolerance capacity, Figure 3(D1,D2) illustrates that the presence of >1280 mg/kg Cd (MTC) completely inhibits the fruiting body growth.

### 3.5. Effect of As Stress on Fruiting Bodies of P. pulmonarius MT

The MTC of Cd is >1280 mg/kg in the substrates of the fruiting bodies of *P. pulmonarius* MT, while the MTC of As is >160 mg/kg (Figure 3(E1,E2)). There is no significant difference in biological yield between any treatment and CK (*p* > 0.05) (0 to 80 mg/kg). When the As dose ranges from 0 to 160 mg/kg in the substrate, the BCF of As decreases from 81.5–106 to 2.7–3.2 (Figure 3(E3)), and the As accumulation capacity is medium (0 to 40 mg/kg) or low (40 to 160 mg/kg).

### 3.6. Remediation Effect of P. pulmonarius MT on Cd- and As-Contaminated Soil

**Figure 4:**
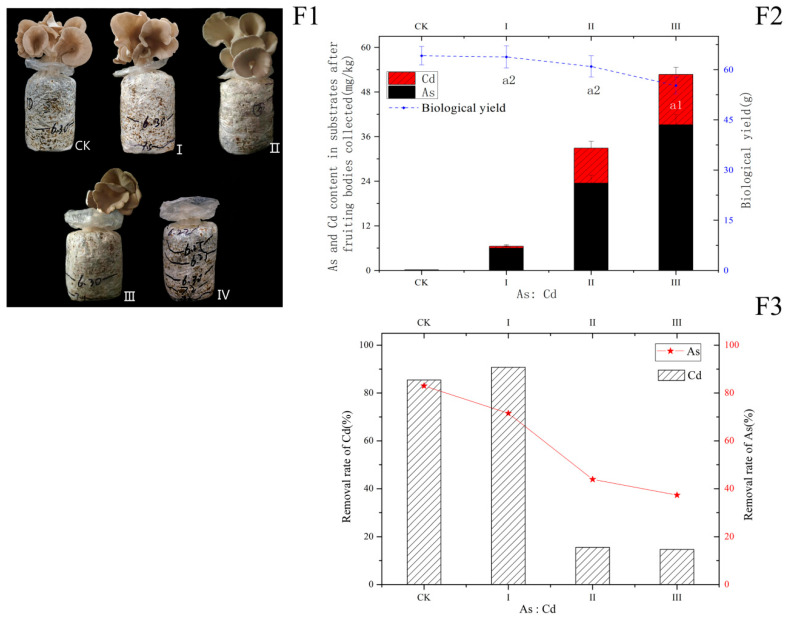
Simulation application in decontamination of soil containing As(III) and Cd(II) by the fruiting bodies of *P. pulmonarius* MT. (**F1**) The growth of fruiting bodies under Cd and As stress; (**F2**) Cd & As content in substrate and the biological yield of fruiting bodies under Cd & As stress; (**F3**) Removal rates of Cd and As. Artificially contaminated soil contained 50 mg/kg of Cd and 200 mg/kg of As(dw). CK: untreated substrates; I: substrates with contaminated soil added by 12% (*w*/*w*, dw); II: 24%; III: 36%; IV: 48%; a1: *p* < 0.05; a2: *p* > 0.05.

Based on the traits of medium As accumulation (0 to 40 mg/kg) and medium As tolerance (MTC > 160 mg/kg) in the fruiting bodies shown above and medium accumulation (0 to 10 mg/kg) and high tolerance to Cd (MTC > 1280 mg/kg), the fruiting bodies of *P. pulmonarius* MT were used to decontaminate As- and Cd-contaminated soil. When the As and Cd concentrations in contaminated substrates are 24 and 6 mg/kg, the As and Cd removal rates are 71.51% and 90.79%, respectively (Figure 4(F3)). Moreover, the fruiting body growth is not significantly inhibited compared with that of CK (*p* > 0.05, Figure 4(F1,F2)). The strain for treatment II can take up about 15.58% of Cd and 43.89% of As of the substrates into the fruiting bodies, and the biological yield is not significantly different from that of CK (*p* > 0.05). Meanwhile, as shown in Figure 4(F1) II and III, the thickness of the pileus reduces, and the color changes from drab yellow to gray in fruiting bodies under treatment II (48 and 12 mg/kg for As and Cd, respectively) and treatment III (72 and 18 mg/kg for As and Cd, respectively), compared with those of CK treatment. This phenomenon is perhaps related to the toxic effect of the TTEs or nutritional deficiency. However, the results demonstrate that intoxication symptoms will not appear in the fruiting bodies under Cd of 10–20 mg/kg or As of 40–80 mg/kg (Figure 3(D1,E1)). We speculate that nutritional deficiency is the main cause of this phenomenon.

## 4. Discussion

Mycoremediation is of considerable interest as a branch of low-cost and eco-friendly technology in contaminated aquatic systems and soil. In recent years, researchers have found some wild fungi resources with high- or hyper-accumulation capacity of TTEs, for example, *Amanita strobiliformis* and *Suillus luteus* [28,29]. Through biological techniques, researchers have also acclimated or bred some fungi strains in labs, including *Pleurotus ostreatus* HAU-2 [21] and *Lentinula edodes* W1 [30,31]. The non-MT Cd-binding protein from *Lentinula edodes* (LECBP) was investigated as a potential remediation tool for Cd biosorption in *Escherichia coli*. This research not only sheds light on the potential of LECBP for Cd bioremediation, but also contributes to a better understanding of the relationship between LECBP’s structure and functionality. Also, several studies focused on the influence factors and mechanisms of accumulation and tolerance [32,33,34].

It seems that most microorganism strains feature extreme accumulation and tolerance capacities to TTE (i.e., high accumulation and low tolerance, low accumulation and high tolerance, or low accumulation and low tolerance). Despite these advantages, the most important question is how to improve the accumulation and tolerance capacities of fungi, and obtaining a fungus strain featuring the two traits at reasonable degrees is important for environmental applications [35,36]. It is a slight possibility that these microorganisms will be applied for TTE removal until researchers find a microorganism strain that simultaneously has high accumulation and medium tolerance capacities or medium accumulation and high tolerance capacities. This paper aims to evaluate the TTEs tolerance and accumulation traits of a new strain *P. pulmonarius* MT to obtain a strain with TTEs high accumulation and medium tolerance or with TTEs medium accumulation and high tolerance.

TTEs are among the major pollutants discharged by urban and agricultural runoffs, industrial effluents, mining, and other processes. Various TTEs such as Pb, Mn, As, Al, Cr, Cd, Co, Cu, Zn, Ni, and Fe have been detected in coal washery effluents [16]. In most cases, two or more kinds of TTEs always exist in environmental pollutants. It is necessary to explore some efficient techniques or fungi for removing two or more kinds of TTEs. A fungi strain that can simultaneously remove two or more kinds of TTEs in pollutants has rarely been reported. *Laccaria bicolor* has the potential to ameliorate the effects of non-essential Cd and essential Cu through different blocking strategies [37]. In one study, the fungus *Pleurotus ostreatus* HAU-2 could remove Cd and Cr in liquid culture through absorption [21]. Our study aims at obtaining an efficient fungus for decontaminating pollutants containing Cd and As.

The application potential of Basidiomycota, including *Pleurotus*, an important edible mushroom, in TTEs accumulation has started to draw increasing attention. *Pleurotus ostreatus* has been reported to have promising applications for removing TTEs from washery effluent and absorbing Cd and Cr from soil [16,21]. Another study provided insights into the transcriptional response of *Pleurotus eryngii* to extremely high levels of HMs [26]. In Q. Li’s study, the upregulation of genes encoding putative oxidoreductases, dehydrogenases, reductases, transferases, and transcription factors following exogenous NO induction contributed to the increased tolerance of *P. eryngii* to high levels of HMs. This study sheds new light on the transcriptional response of *P. eryngii* to HMs and the role of NO in improving heavy metal tolerance. In our next study, we will attempt to delve into researching how *P. pulmonarius* MT responds to extremely high concentrations of TTEs stress by transcriptomics. One study evaluated the feasibility of removing Cu(II) and Zn(II) using ahybrid immobilized biosorbent of *Pleurotus sajor-caju* and *Jasmine sambac* [14].

Cihangir and Saglam observed that the Cd uptake rate by *Pleurotus sajor-caju* onto dry biomass was between 88.9% and 91.8% using aqueous media with concentrations ranging from 0.125 to 1.0 mM (about 14–112 mg/L), and this strain might successfully be utilized for the removal of TTEs in polluted water with 0.5 mM Cd^2+^ (about 56 mg/L) [38]. In this study, when the concentrations of Cd and As were 20 and 80 mg/L, respectively, the Cd removal rate was about 98.99%, and 15.43% of As was removed; moreover, the hypha growth was not significantly inhibited. The trend of Cd accumulation in PDB in our research almost accords with that of Cihangir and Saglam’s report. The abilities of some filiform fungi, ectomycorrhiza, and yeast for TTEs removal from liquid and solid media have been confirmed [39,40,41]. It has been suggested that the hypha of *P. pulmonarius* MT might successfully be used in processes related to the removal and recovery of TTEs from aqueous pollutants.

Edible mushrooms including *P. pulmonarius* MT have a striking advantage over other microorganisms as an efficient method of mycoremediation. This reflects a high biological efficiency of edible mushrooms. For example, the biological efficiency of *P. pulmonarius* MT is about 70%. In other words, the whole weight of 1 kg (dw) substrates used to cultivate *P. pulmonarius* MT resulted in a total biological yield of fruiting bodies of generally 0.70 kg (fw) after the harvest of three or four batches. This explains an unusual point in Figure 4(F3), why the removal rate of Cd (90.79%) is higher than that of As (71.51%) with As and Cd concentrations of 24 and 6 mg/kg in contaminated substrates, respectively; however, there is no large difference in the accumulation capacity between 20 mg/kg for As treatment (Figure 3(E1)) and 5 mg/kg for Cd treatment (Figure 3(D1)).

## 5. Conclusions

The hypha of *P. pulmonarius* MT cultivated in PDA exhibit medium or high Cd accumulation (0 to 320 mg/L), medium Cd tolerance (MTC ≥ 640 mg/L), medium As accumulation (0 to 80 mg/L), and high As tolerance (MTC >1280 mg/L). Moreover, the hypha of *P. pulmonarius* MT was utilized in processes related to the removal of Cd and As present in aqueous pollutants at concentrations of 80 mg/L and 20 mg/L, respectively. The trends for the fruiting bodies of *P. pulmonarius* MT seem to deviate from those of the hypha of this strain. The results show that the fruiting bodies feature medium As accumulation (0 to 40 mg/kg), medium As tolerance (MTC > 160 mg/kg), medium Cd accumulation (0 to 10 mg/kg), and high Cd tolerance (MTC > 1280 mg/kg). The fruiting bodies were further utilized in processes related to the recovery of Cd and As in substrates, that is, 12% contaminated soil mixed with 50 mg/kg Cd and 200 mg/kg As. According to the results, *P. pulmonarius* MT is a promising candidate for the remediation of As- and Cd-contaminated soil or water. Nevertheless, there are still some challenges in understanding the remediation mechanism of this strain, recycling the TTEs from contaminated hypha or fruiting bodies, and fully utilizing residuals.

## Data Availability

Experimental details relating to this paper are available online.
